# Case report: two novel *VPS13B* mutations in a Chinese family with Cohen syndrome and hyperlinear palms

**DOI:** 10.1186/s12881-019-0920-x

**Published:** 2019-11-21

**Authors:** Sha Zhao, Zhenqing Luo, Zhenghui Xiao, Liping Li, Rui Zhao, Yongjia Yang, Yan Zhong

**Affiliations:** 1 The Laboratory of Genetics and Metabolism, Hunan Children’s Research Institute (HCRI), Hunan Children’s Hospital, University of South China, Changsha, 410007 China; 2 Department of Child Healthcare, Hunan Children’s Hospital, University of South China, Changsha, 410007 Hunan China

**Keywords:** Cohen syndrome, Hyperlinear palm, VPS13B gene, Mutation, Chinese

## Abstract

**Background:**

Cohen syndrome (CS) is an uncommon developmental disease with evident clinical heterogeneity. *VPS13B* is the only gene responsible for CS. Only few sporadic cases of CS have been reported in China.

**Case presentation:**

A Chinese family with two offspring–patients affected by developmental delay and intellectual disability was investigated in this study. Exome sequencing was performed, and compound heterozygous mutations in *VPS13B* were segregated for family members with autosomal recessive disorder. Splicing mutation c.3666 + 1G > T (exon 24) and nonsense mutation c. 9844 A > T:p.K3282X (exon 54) were novel. We revisited the family and learned that both patients are affected by microcephaly, developmental delay, neutropenia, and myopia and have a friendly disposition, all of which are consistent with CS phenotypes. We also found that both patients have hyperlinear palms, which their parents do not have. *VPS13B* mutations reported among the Chinese population were reviewed accordingly.

**Conclusions:**

This study presents two novel *VPS13B* mutations in CS. The identification of hyperlinear palms in a family affected by CS expands the phenotype spectrum of CS.

## Background

Cohen syndrome (CS) (MIM# 216550), a rare disorder, was initially described by M. Michael Cohen, Jr., and his colleagues in 1973 [1]. CS is a clinically heterogeneous disorder mainly characterized by developmental delay, intellectual disability, microcephaly, and hypotonia with highly variable clinical findings on myopia, retinal dystrophy, joint hypermobility, neutropenia, overly friendly behavior, truncal obesity, slender fingers, and facial appearance consisting of thick hair, thick eyebrows, long eyelashes, down-slanting eyes, short philtrum, and prominent upper incisors [2].

The clinical heterogeneity and several phenotypes of CS are unobservable before 10 years old; thus, this rare disease is difficult to diagnose in clinical practice. Nevertheless, CS can now be diagnosed through *VPS13B* mutation screening with the cloning of the disease-causing gene *VPS13B* [3–5]. *VPS13B* is the only gene that causes CS. Several *VPS13B* mutations have been recently reported in families with CS in Tunisia and Pakistan [6, 7].

Human *VPS13B* (NM_017890), which is located on 8q22.2, consists of 62 exons that encode a 4022-amino acid transmembrane protein of the Golgi apparatus functioning in vesicle-mediated transport and sorting of proteins within the cell [3]. Approximately 200 *VPS13B* mutations (http://www.hgmd.cf.ac.uk/) have been reported in nearly 1000 CS patients worldwide (http://www.cohensyndrome.org/). Founder mutations have been described in several areas [2]. Only a few *VPS13B* mutations have been reported sporadically in patients with CS (two definitive CS and three probable CS) in the Chinese population [8–10]. In the present study, exome sequencing identified two novel *VPS13B* mutations in a Chinese family with two offspring–patients affected by CS and hyperlinear palms. The presence of hyperlinear palms can likely expand the phenotypic spectrum of CS.

## Case presentation

The study protocol was approved by the Academic Committee of Hunan Children’s Hospital (Approval No. HCHLL58, Changsha City, Hunan Province, China). The four family members (Han Chinese ethnicity), which included two parents and two children, provided written informed consent to participate in this study. Parental permission for publishing patients’ photos was obtained. The genomic DNAs of all four family members were isolated using standard methods.

### Patient 1

**Fig. 1:**
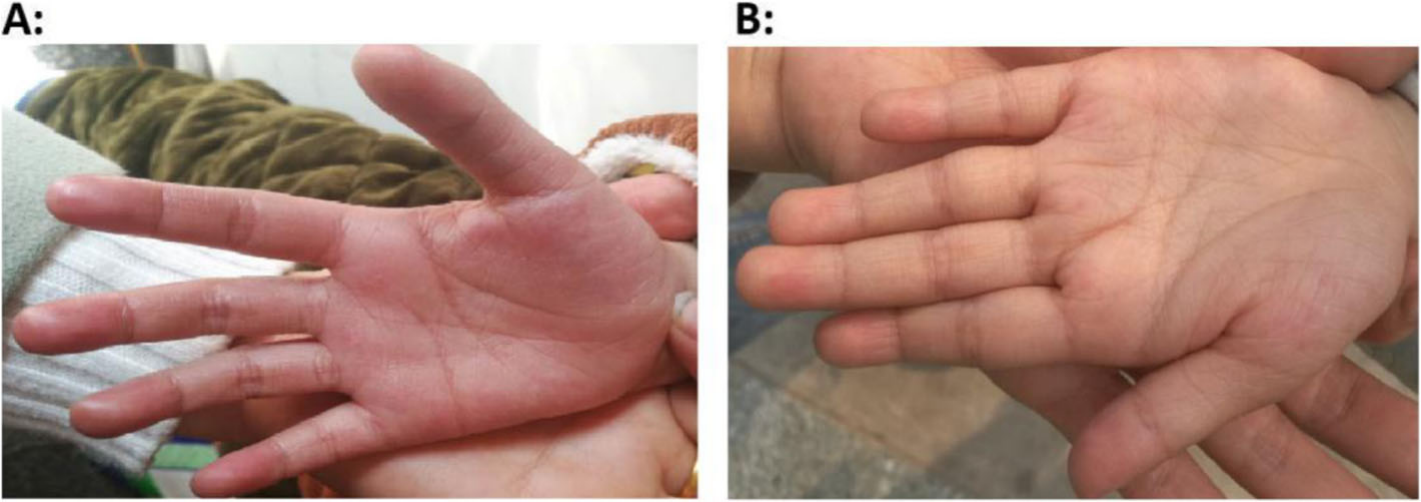
Patients with CS in a Chinese family. **a** Left hand palm of female patient M2651 and **b** Right hand palm of male patient M2650

The propositus was the first child born to the healthy nonconsanguineous parents (father’s age: 27; mother’s age: 22) after a spontaneous uncomplicated delivery with Apgar scores of 9 and 10. The patient’s birth weight was 2900 g (25th–50th centile), and his length was 48.5 cm (25th–50th centile). He was evaluated when he was 7.5 years old with a height of 111.4 cm (− 3.02 SD), weight of 23.5 kg (25th–50th centile), and occipitofrontal circumference (OFC) of 45.2 cm (normal reference: 52.6 cm). The patient has been walking alone since he was two years old, but he cannot complete a sentence (i.e., more than two words). The patient exhibits hyperactivity, disobedience, severe mental retardation, generalized joint hyperextensibility, hypotonia, slender fingers, hyperlinear palm (Fig. 1a), and special facial features, including prominent upper central incisors, thick hair, thick eyebrows, micrognathia, prominent upper lip, and prominent root of the nose. Neutropenia (neutrophil count: 0.58 × 109/L, normal range: 2.00–7.00 × 109/L) was observed when the patient was 8.2 years old and occurred after a cold. Truncal obesity was unobservable.

### Patient 2

The propositus’ younger sister was evaluated when she was 5.8 years old. Her height was 97.5 cm (− 3.72 SD), her weight was normal, and her OFC was 43.5 cm (normal reference: 51.5 cm). She has been walking alone since she was 2.5 years old, but she cannot complete a sentence. Similar to her older brother, she exhibits severe mental retardation, generalized joint hyperextensibility, and hypotonia. Her special facial features include prominent upper central incisors, thick hair, thick eyebrows, long and thick eyelashes, bilateral ptosis, bilateral epicanthus inversus, and bilateral strabismus. Neutropenia and truncal obesity were unobservable. The patient has hyperlinear palms (Fig. 1b).

### Genetic testing

**Fig. 2:**
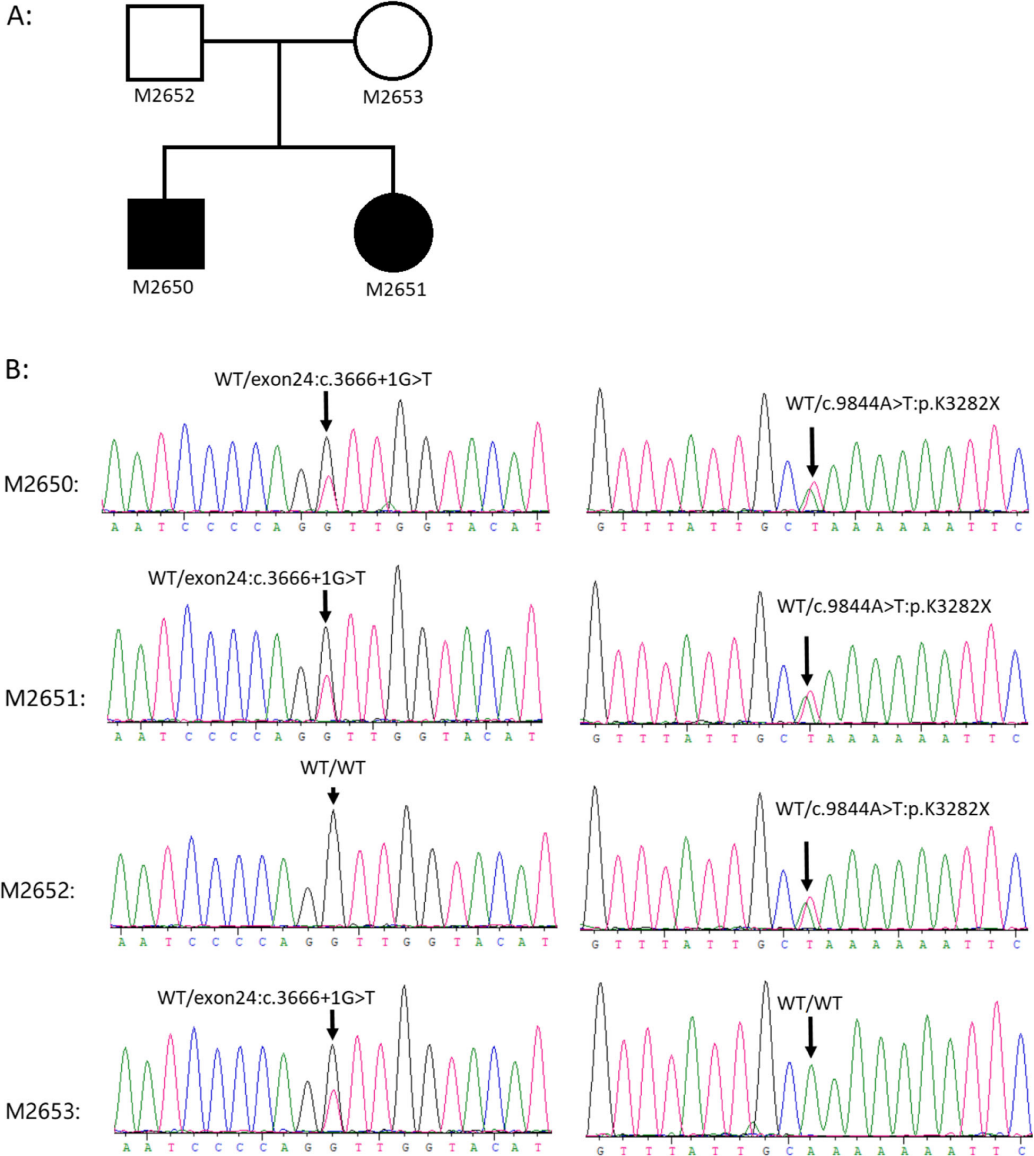
*VPS13B* mutations in a Chinese family with CS. **a** Pedigree. **b** Chromatograms of *VPS13B* mutations by Sanger sequencing

CS in the Chinese population has rarely been reported. Neutropenia was unobservable in the selected family before this experiment. GTG banding was initially performed, and the karyotypes of the four family members were normal. Then, a copy number variation (CNV) test for the propositus (M2650, Fig. 2a) was performed with the Illumina Human OmniZhongHua-8 BeadChip array (Illumina, San Diego, CA, USA). No pathogenic CNV was detected, and the family likely suffered from a monogenetic disease. Exome sequencing of the four members was performed according to a previously described pipeline [11]. No consanguineous marriage in the family was reported. The severe phenotypes of both offspring (but none of the parents) exhibited abnormalities. We focused on genes/variants that met the following criteria: (1) coding region variants with MAF < 0.001 (All_gnomAD, ExAc, 1000 genomes, and ESP6500); (2) damaging variants consisting of loss-of-function (LOF) and damaging missense variants (VarCards); (3) recessive inheritance gene consisting of compound heterozygous or homozygous variants; and (4) the gene function related to the phenotypes. Only one gene (compound heterozygous variant), that is, *VPS13B* (data not shown), was selected. Sanger sequencing was performed (the primer and PCR condition are provided in Additional file 1: Table S1). The two LOF variants of *VPS13B* (chr8:100479863G > T, NM_017890-c.3666 + 1G > T and chr8:100847793A > T, c.9844A > T:p.K3282X) were confirmed, and the *VPS13B* variants were co-segregated with the disorder in autosomal recessive mode in the family (Fig. 2b).

## Discussion and conclusions

A family with two offspring–patients affected by severe mental retardation and speech delay came to our clinic for genetic counselling. The detection of compound heterozygous mutations in *VPS13B* via exome sequencing directed us to the diagnosis of CS in the family. CS is a clinical heterogeneous disorder whose occurrence is high among patients with different ethnic backgrounds [12]. For example, patients who belong to Finnish and Greek cohorts present much richer skeletal phenotypes featured by slender extremities and/or tapered fingers, joint hypermobility, and sandal gap compared with patients who belong to the same cohort [12, 13].

The two patients with CS in this study have slim or slender fingers. Hyperlinear palms were also observed in the two patients but not in their parents and other family members (Fig. 1). Hyperlinear palms or palms with extra skin creases signify a hand anomaly (“https://www.rightdiagnosis.com/symptom/hyperlinear-palms-extra-skin-creases-in-the-palms.htm”). Hyperlinear palms have been reported among *FLG*-mutated patients [14] with atopic dermatitis or ichthyosis vulgaris [15]. We tried contacting *VPS13B*-mutated Chinese patients [8–10] to see if they have hyperlinear palms as a means of verifying if hyperlinear palms are a component phenotype of CS. However, we have not received any response from these patients. Therefore, additional supporting data are required to determine if hyperlinear palms are a CS phenotype.

**Fig. 3:**
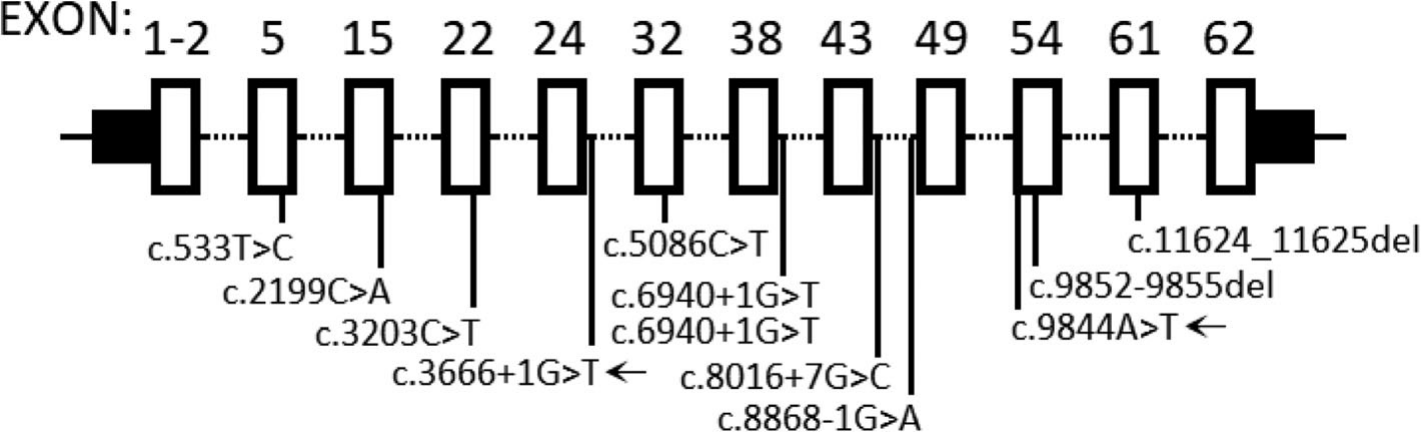
Schematic presentation of *VPS13B* mutations in the Chinese population. The arrow represents the mutations identified in the present study

CS was initially described in 1973 [1], and *VPS13B* mutation in CS was reported in 2003 [3]. However, only two definitive CS [8, 9] and three probable CS patients have been reported in the Chinese population; the first patient with CS was diagnosed by next-generation sequencing in 2016 [8]. In the present study, we reviewed all *VPS13B* mutations reported in the Chinese population (Fig. 3). Only one *VPS13B* mutation (c.6940 + 1G > T) was reported twice, and the others in the *VPS13B* gene were evenly distributed. Further studies involving Chinese patients with CS are needed to determine if founder mutation of *VPS13B* occurs among Chinese families.

This study identified two novel *VPS13B* mutations in a Chinese family with two members affected by CS. This work is the first to report a complete set of CS data involving a Chinese family. The presence of hyperlinear palms (identified in this study) is likely a novel phenotype of CS and may provide new clues for CS diagnosis.

## Supplementary information

**Additional file 1: Table S1.** Primers and PCR conditions in this study.

## Data Availability

All data generated or analyzed during this study are included in this published article.

## Abbreviations

CNV: Copy number variation
CS: Cohen syndrome
LOF: Loss of function
OFC: Occipitofrontal circumference
PCR: Polymerase chain reaction
