# Supplementary material for: Case report: two novel VPS13B mutations in a Chinese family with Cohen syndrome and hyperlinear palms
Source: BMC Med Genet. 2019 Nov 21;20:187. doi: 10.1186/s12881-019-0920-x (PMC6873578; doi:10.1186/s12881-019-0920-x)
Supplement: Supplementary file 1 — Additional file 1: Table S1. Primers and PCR conditions in this study. [file 12881_2019_920_MOESM1_ESM.docx]

**Additional file 1: Table 1.**

Primers and PCR conditions used in the present study.

| **Gene(exon)** | **Forward** | **Reverse** | **Product  size** | **Annealing  temperature** |
| --- | --- | --- | --- | --- |
| VPS13B(24) | 5'-cttggacgatcagcttgcat-3' | 5'-accctgatcttgatggccaa-3' | 342bp | 60°C |
| VPS13B(54) | 5'-agtagttacctgtgatcctgtga-3' | 5'-aggttcaactctcaaaagtaggc-3' | 289bp | 60°C |
